# Perioperative Assessment and Clinical Outcomes of Percutaneous Atrial Septal Defect Closure with Three-Dimensional Transesophageal Echocardiography

**DOI:** 10.3390/diagnostics14161755

**Published:** 2024-08-12

**Authors:** İbrahim Saraç, Oğuzhan Birdal

**Affiliations:** 1 Department of Cardiology, Faculty of Medicine, Atatürk University, Erzurum 25010, Türkiye; droguzhanbirdal@gmail.com; 2 Department of Physiology, Faculty of Medicine, Atatürk University, Erzurum 25010, Türkiye

**Keywords:** imaging, atrial septal defect, three-dimensional transesophageal echocardiography, two-dimensional transesophageal echocardiography, percutaneous closure, structural heart disease

## Abstract

Background/Objectives: To close the atrial septal defect (ASD) with the transcatheter method, correctly defining the defect and selecting the appropriate closure device size are the most critical steps for the procedure’s success. Although ASD can be successfully closed under the guidance of three-dimensional (3D) transesophageal echocardiography (TEE) and two-dimensional (2D) TEE, measurement comparisons between different types of defects are still needed. Methods: Our study was designed retrospectively. Sixty-one patients who underwent transcatheter ASD closure with 2D TEE and 3D TEE between 2020 and 2024 were included. The patients were divided into three groups according to the defect shape: circular, oval, and complex; and the measurement results, perioperative process, and clinical outcomes were compared in each group. Results: The average age of the patients was 35.05 ± 13.87 years, and 41 (67.2%) were women. The average follow-up period of the patients was 15.3 ± 9.18 months. No statistical significance was observed in the comparison of measurements obtained with 3D TEE and 2D TEE in the circular and oval defect groups. The differences between the minimum defect diameters of complex defects measured by 2D TEE and 3D TEE (*p*: 0.037), IVC rims (*p* < 0.001), aortic rims (*p*: 0.012), and the differences between implanted device dimensions and the maximum defect diameters measured by both methods were compared; statistical significance was observed (*p*: 0.025). Conclusions: In circular and non-complex oval defects, it has been observed that the size of the closure device selected with 2D TEE or 3D TEE is optimal, and the procedure is practical and feasible. While the closure of complex ASDs with 3D TEE provides reliable and optimal results, using only 2D TEE in complex ASDs may lead to selecting a smaller-sized device.

## 1. Introduction

Atrial septal defect (ASD) is the most common congenital heart disease in adults [1]. ASD closure is indicated in symptomatic patients or in asymptomatic patients with left-to-right shunt who have right heart dilatation due to increased volume load. Closure of small defects without right ventricular volume overload is not recommended unless there is evidence of hypoxemia and/or paradoxical embolism [2,3,4]. While ASD treatment was previously performed surgically, today, it is successfully applied with a low complication rate because of the techniques and devices developed for transcatheter treatment [3,4,5]. Since the interatrial septum (IAS) has a very dynamic and complex three-dimensional anatomical structure, it is very important to accurately define the defect’s anatomy to select the appropriate device when transcatheter closure is decided [6]. The balloon sizing (BS) method was previously used commonly in determining the defect and device diameter in transcatheter closure of ASD. Still, some of its limitations and complications are known [7]. Excessive stretching of the residual rims during defect diameter measurement with BS may cause an increase in the selected device size, rupture of the septum, cardiac rupture, cerebral micro embolism, and balloon rupture. In addition, BS may cause increased radiation exposure and prolong procedure time [8,9]. Although two-dimensional (2D) transesophageal echocardiography (TEE) is widely used to evaluate ASD, it cannot provide clear information about the spatial location and anatomy of the defect [9,10,11]. Three-dimensional (3D) TEE is useful for evaluating ASD and its relationship with important structures surrounding it (ASD’s type, size, shape, orientation, number, location, and rim presence) [4,11,12,13]. In many studies comparing 3D TEE and BS, perioperative 3D TEE is effective and safe in ASD closure; it has been emphasized that ASD can be closed with 3D TEE without using BS [14,15,16]. Although many studies are comparing 3D TEE, BS, and 2D TEE measurements, comparisons of measurements in different types of defects are still needed [17,18]. In this study, defect anatomy and dimensions in patients with circular, oval, and complex-shaped ASD were evaluated with 2D TEE and 3D TEE. These measurement methods compared implanted device dimensions and perioperative and late-term clinical results.

## 2. Materials and Methods

### 2.1. Patients Selection

**Figure 1:**
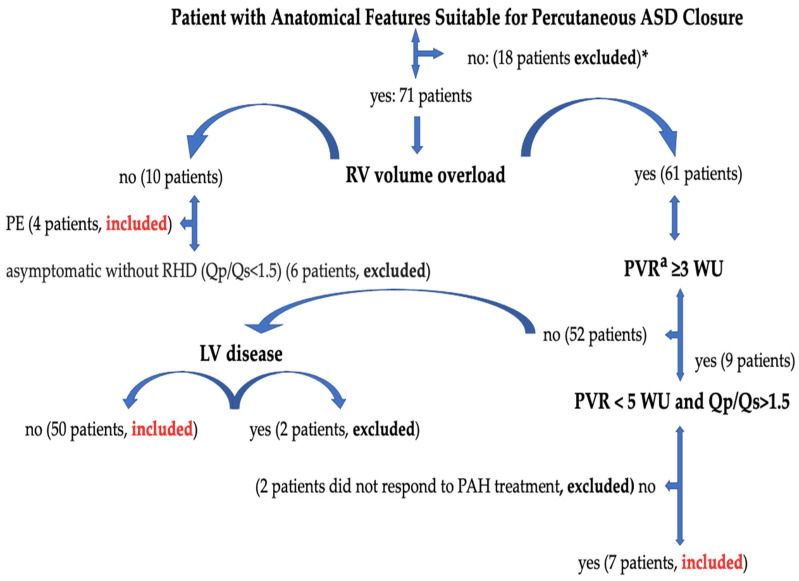
Management of atrial septal defect and patient selection. ASD, atrial septal defect; PE, paradoxical embolism; PVR, pulmonary vascular resistance; RV, right ventricle; LV, left ventricle; RHD, right heart dilatation; Qp/Qs, pulmonary-to-systemic flow ratio; WU, wood units. *: Ostium primum ASDs, sinus venosus ASDs accompanied by unroofed coronary sinus and abnormal pulmonary venous return, complex secundum ASDs accompanied by an overly flaccid aneurysmatic septum, patients with multiple defects that are not suitable for percutaneous closure, valves that require additional surgery, patients with congenital and coronary pathology, and ASDs with missing rims other than the aortic rim (less than 5 mm) were not suitable for transcatheter closure and were referred to surgery. **a**: Cardiac catheterization is required in case of non-invasive signs of PAP elevation (calculated systolic PAP > 40 mmHg or indirect signs when PAP cannot be estimated) to determine PVR. Right heart catheterization was performed in 40 patients with sPAP values above 40 mmHg. 2 patients were not included in the study [3].

Sixty-one patients who underwent percutaneous ASD closure with 2D/3D TEE guidance at Erzurum City Hospital between 2020 and 2024 were included in the study. In patient selection, the European and American Society of Cardiology Adult Congenital Heart Diseases Guidelines’ recommendations were considered. A percutaneous ASD closure device was implanted in symptomatic patients with a pulmonary-to-systemic shunt ratio (Qp/Qs > 1.5) or in patients with increased right ventricular volume load on transthoracic echocardiography (TTE). Right heart catheterization was performed in patients with TTE, which showed increased right ventricular volume and systolic pulmonary arterial pressure (sPAP). The decision to close percutaneous ASD was made for patients whose pulmonary vascular resistance (PVR) was invasively confirmed to be below three wood units (WU) or in patients whose PVR was between 3 and 5 WU and had a significant L–R shunt (Qp:Qs > 1.5). In patients with evidence of paradoxical embolism (although other medical causes were excluded), it was decided to close the ASD, regardless of symptoms and shunt rate. Ostium primum ASDs, sinus venosus ASDs accompanied by unroofed coronary sinus and abnormal pulmonary venous return, complex secundum ASDs accompanied by an overly flaccid aneurysmatic septum, patients with multiple defects that are not suitable for percutaneous closure, valves that require additional surgery, patients with congenital and coronary pathology, and ASDs with missing rims other than the aortic rim (less than 5 mm) were not suitable for transcatheter closure and were referred to surgery. ASD was not closed in patients with Eisenmenger physiology, in patients with PVR ≥ 5 WU despite treatment for pulmonary arterial hypertension (PAH), and in patients with left ventricular systolic heart failure [3,4]. Management of atrial septal defect and patient selection are shown in Figure 1.

### 2.2. 2D TEE and 3D TEE Procedure and Image Optimization

**Figure 2:**
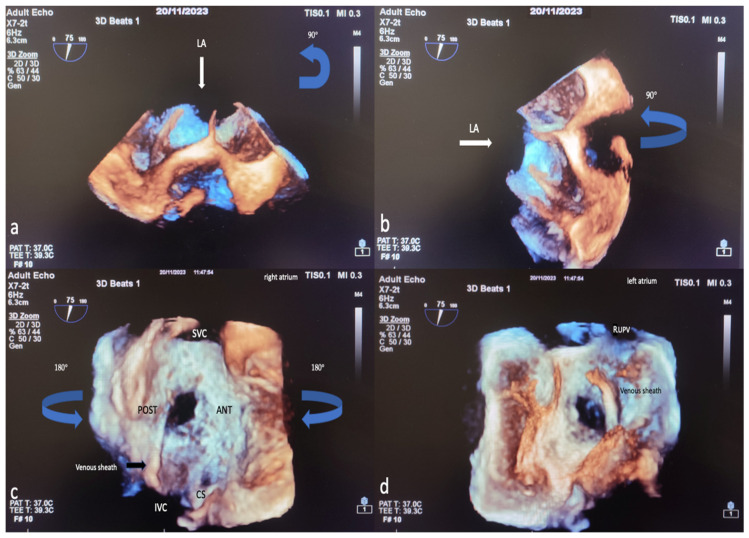
Image of percutaneous ASD closure process with 3D TEE, image optimization in 3D TEE. In 2D TEE examination, when the bicaval window (when examining the SVC-IVC plane section at approximately 80–120 degrees) is switched to 3D zoom examination, the Image on the monitor is obtained with the transducer facing the left atrium perpendicularly from above (**a**). The operator first views the Image 90 degrees counterclockwise (blue arrow) (**a**) in the sagittal axis and then rotates 90 degrees to the left on the vertical axis (blue arrow) (**b**) to obtain the right atrial view (**c**). After obtaining the RA (en face) view in this way, the LA (en face) view will be obtained by rotating 180 degrees in the vertical axis to the right or left (**d**). Based on these LA and RA facial views, ASD morphology and the status of the rims are easily identified. LA, left atrium; ANT, anterior; SVC, superior vena cava; POST, posterior; IVC, inferior vena cava; CS, coronary sinus; RUPV, right upper pulmonary vein; 2D, two-dimensional; 3D, three-dimensional. (The images included in this article were taken from Erzurum City Hospital Cardiology Clinic Echocardiography laboratory).

The TEE procedure was performed under local infiltration oropharyngeal anesthesia with lidocaine mucilage in patients who tolerated it. For patients who could not tolerate it, TEE was performed under sedation. All patients were fasted for 12 h before TEE. During the procedure, vital signs such as heart rate, blood pressure, electrocardiography (ECG), and fingertip blood oxygen saturation were monitored. All 2D TEE and 3D TEE imaging was performed using the Philips EPIQ7 Cardiac Ultrasound System and the X7-2t (Bothell, WA, USA, Ultrasound Transducer) TEE probe. The TEE procedure was performed according to the American Society of Echocardiography (AED)’s standard protocol [19]. Initially, ASD diameters, shapes, and morphologies were visually examined and measured with 2D TEE in the evaluation made from four-chamber (0°), short axis (30–60°), bicaval axis (90–120°) and 0–180° angles. Measurement of the minimum and maximum diameters of the ASD was obtained by routine angular and rotational (counterclock vise) movement of the 2D TEE probe between 0 and 180°. In 2D ASD imaging, the maximum and minimum diameters were measured and recorded when the defect sizes appeared largest at the end of the cardiac systolic phase. Images are routinely taken by the recommendations in the 3D echocardiography guide. A sufficient 3D TEE imaging dataset was collected by recording 3D full volume, Live 3D, 3D zoom, X plane (multiplane, biplane), and multiple-beat 3D imaging samples volume images accompanied by ECG [20]. The first image obtained when switching from a mid-esophageal bicaval examination in 2D TEE to 3D TEE zoom mode is shown in Figure 2a. The image in Figure 2b is obtained by rotating the first image 90 degrees counterclockwise. The images of RA and LA (en face), respectively, obtained by 3D TEE zoom mode imaging are shown in Figure 2c,d. The EAE/ASE recommendations for image acquisition and display using three-dimensional echocardiography practice guidelines are the basis for image acquisition, display, and image optimization. Generally, gain and compression settings were set in the midrange and optimized with slightly higher time gain controls (time gain compensation) to enable the greatest flexibility with postprocessing gain and compression [11]. Two experienced operators performed the TEE procedure, and two experienced interventional cardiologists performed ASD closure.

### 2.3. Data Analysis

**Figure 3:**
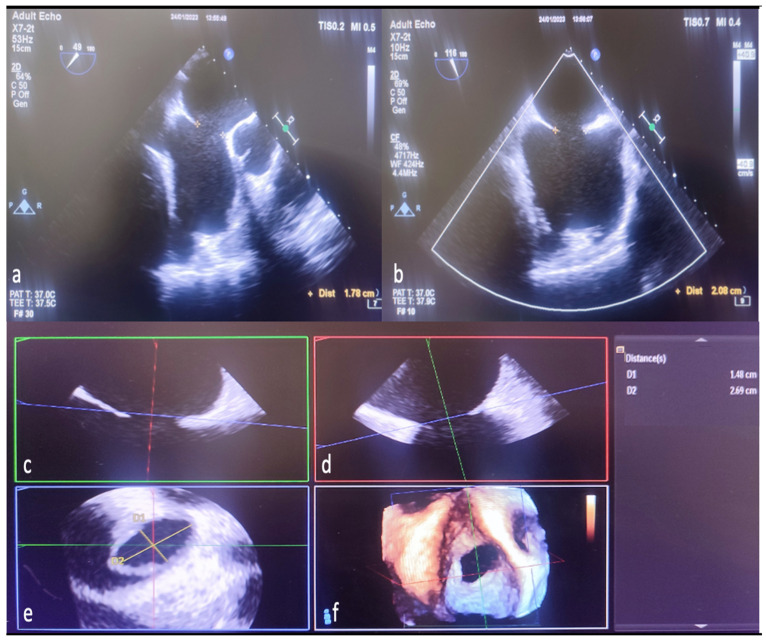
Measurement of defect diameters with MPR in 3D TEE and comparison with 2D TEE. The length ASD of the was measured as 1.78 cm on 2D TEE mid-esophageal short-axis imaging (**a**). The same defect was measured as 2.08 cm in bicaval examination with 2D TEE (**b**). Display ASD diameters in multiple planes to generate MPR from 3D TEE zoom mode examination datasets (**c**,**d**). Length measured from the anteroposterior (short-axis-minimum diameter) aperture plane (red line) in multi-planer examination (1.78 cm defect measured in 2D TEE) (**c**,**e**,**f**). Again, the specified length (measured by 2D TEE) from the superoinferior (bicaval-long-axis-maximum diameter) aperture plane (green line) is seen as 2.08 cm (**d**–**f**). Naturally, in the final image created with MPR as a result of combining the planes (indicated by red, green, and blue lines), it is seen that the defect is ellipsoid, and the real minimum diameter (D1 yellow line-short axis) is 1.48 cm, the real maximum diameter (D2 yellow line-long axis) is 2.69, measured in cm (**e**). MPR, multiplanar reconstruction; 2D, two-dimensional; 3D, three-dimensional.

The analysis of 3D image data was performed offline in accordance with the guidelines [11]. Since the shape and size of the defect change dynamically throughout the cardiac cycle, both minimum and maximum ASD aperture diameters were measured at the end of the systole in the 2D ASD imaging. In addition, residual rims in 3D were obtained in the face view at the end of the systole during the largest defect opening. In addition, cropping (auto crop and iCrop) was performed to determine the measurement, axis, and localization of some rims. Defect diameters were measured offline with the multiplanar reconstruction (MPR) method from 3D zoom datasets in QLAB (QLAB version 10.5 software (Philips Medical System, Best, The Netherlands). The mid-esophageal short- and long-axis examination of ASD by 2D TEE is shown in Figure 3a,b. Reconstruction of different planes in 3D TEE multiplanar examination shown in Figure 3c,d. The measurement of ASD diameters by MPR method in the 3D TEE examination is shown in Figure 3e,f.

**Figure 4:**
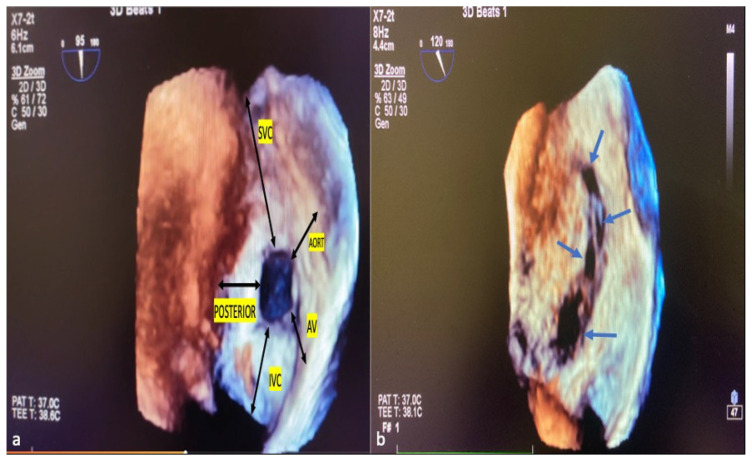
ASD rims and multi-fenestrated ASD image in 3D TEE. The tissue lengths indicated by black arrows in the RA (**a**), en face view, describe the ASD rims. Example of complex, multi-fenestrated ASD (Blue arrows) (**b**). SVC, superior vena cava; AV, atrioventricular; IVC, inferior vena cava; POSTERIOR, posterior rim; AORT, aortic rim.

ASD residual rims: This is defined as the distance between the edges of the ASD and the surrounding intact atrial septum tissue. ASD residual rims were examined in detail by slicing or cropping 3D full-volume and 3D zoom datasets. The maximum rim length was based on the value measured at the end of the systole. The superior or inferior residual rim was determined by the distance from the defect edge to the SVC or IVC in the mid-to-high esophageal position. The posterior rim was measured as the distance between the defect edge and the posterior wall. The antero–superior (aortic) residual tissue rim is the distance between the defect edge and the aortic wall in the mid–upper esophagus position in the short axis. The antero–inferior residual rim (AV valve rim) is the distance between the ASD border and the atrioventricular valve annulus in the mid-esophageal 4-chamber view. ASD morphology: From the en-face view, when the minimum diameter was equal to or greater than 75% of the maximum diameter, it was considered a circular defect, and when it was smaller, it was considered an elliptical or oval defect [21]. Complex ASDs were defined according to their shape and/or location. The ASDs are considered complex if the defects are large (>26 mm), have deficient septal rims (particularly inferior rim), atrial septal aneurysm is present, or defects are multiple or fenestrated [22]. Figure 4a,b show the ASD rims defined, respectively, and a multi-fenestrated ASD example.

### 2.4. Transcatheter ASD Closure and Patient Follow-Up

The procedure was performed under general anesthesia, with TEE guidance and fluoroscopy. All operations were performed as a team with the main operator, assistant operator, and 3D TEE imaging specialist. Anticoagulation was provided for thromboembolic complications with 100 μ/kg heparin during the procedure. The intervention was made through the right femoral vein. A 6F femoral sheath was placed. Then, the guide wire and multipurpose catheter were passed from the ASD into the left atrium, and the guide wire was directed toward the left upper pulmonary vein. Then, the guide wire was exchanged for a stiff wire, and a Mullins-type delivery sheath was placed in the left atrium over the stiff wire. The ASD device was delivered to the left atrium through the sheath via the delivery system. The device was deployed in the atrial septum. In cases where the device was unstable or malformed after implantation, in the presence of moderate or severe residual shunt flow, or in cases where the device was oversized or undersized, the implanted device was removed to the delivery system, and a new device that was optimally suited to the defect was implanted.

**Figure 5:**
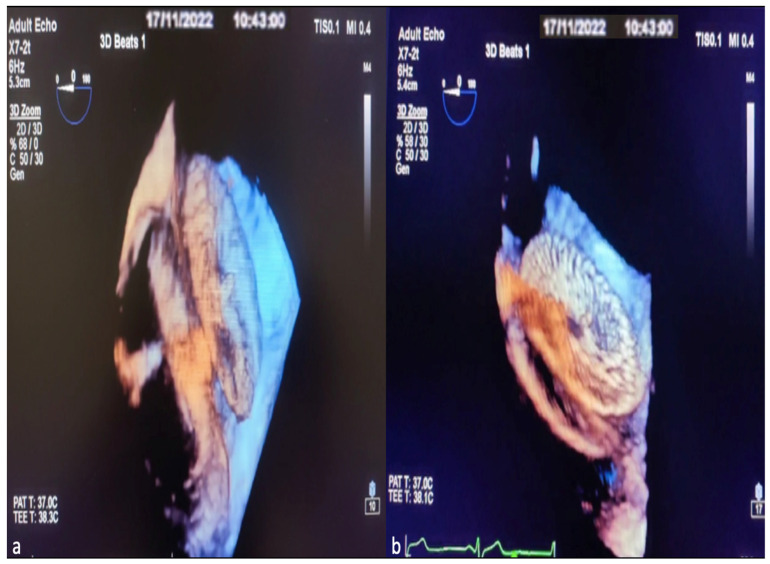
Images of ASD occlusion device closure with intraoperative 3D TEE procedure. View the implanted device from the lateral interatrial septum before it is separated from the delivery system (**a**). Image of the ASD occlusion device after leaving the delivery system (**b**).

Finally, after observing that the device was in the appropriate position and place, the stability of the device and the rims were checked with the Minnesota maneuver (the device is moved back and forth with the help of the catheter). Once the optimal implantation was determined, the device was released, and the procedure was completed. The 3D TEE image of the ASD occlusion device before release and after release is shown in Figure 5a,b.

In our center, where BS was previously performed routinely during the ASD closure procedure, BS was abandoned during the procedure after the 3D TEE experience increased. An Amplatzer Septal Occluder device was implanted in all patients with ASD. Imaging was performed with 2D TTE imaging immediately after implantation, one day later, before discharge, approximately one month after the procedure, six months later, and at long-term follow-up. During this period, data regarding the perioperative-, early-, and late-period clinical follow-ups and outcomes of the patients were obtained from hospital automation, echocardiography laboratory, and fluoroscopy records. The patients were prescribed 100 mg/day or more of aspirin for at least six months after the procedure [23].

### 2.5. Statistical Analysis

Descriptive statistics (number, percentage, mean, standard deviation, median, minimum, and maximum) of the data are stated in the study. As the first step of the statistical analysis, the assumption of normality was checked with the Shapiro–Wilk test, and the homogeneity of variance was checked with the Levene test. Independent Sample T-test was used to compare two independent groups with normal distribution, and the Whitney U test was applied when the normal distribution assumption was not met. Pearson and Spearman’s correlations examined relationships between the maximum size measurements for defect types. ANOVA was used to compare the means of three or more independent groups with normal distribution, and the Kruskal–Wallis test was applied when there was no normal distribution. Analyzes were carried out in the IBM SPSS 25 program.

## 3. Results

Sixty-one patients who underwent percutaneous ASD closure with 3D TEE guidance at Erzurum City Hospital between 2020 and 2024 were included in our study. The average age of the patients was 35.05 ± 13.87 (19–66 years), and 41 (67.2%) of the patients were female. The average follow-up period of the patients was 15.3 ± 9.18 months. Of all the ASD patients, 24 (39.3%) had circular-shaped defects, 17 (27.8%) had oval-shaped defects, and 20 (32.7%) had defects that were complex in shape. The primary indication for ASD closure was right ventricular dilation with pulmonary hypertension, symptoms, or the presence of a significant shunt in 48 patients (78.6%), symptoms in the absence of right ventricular dilation in 9 patients (14.7%), and stroke or transient ischemic attack from presumed paradoxical embolism in four patients (6.65%). Right heart catheterization was performed in 38 (63.9%) patients whose ASD was closed. The baseline characteristics of the 61 study patients are shown in Table 1.

In the complex-shaped group, the selected device was oversized in 1 patient and undersized in 1 patient, and the device was replaced with the optimal device without abandoning it. In the oval-shaped defect group, the device was observed to be oversized in 2 selected patients and was replaced with the optimal device without being released. Again, in the patient group with complex ASD, in a patient without an aortic rim, the inferior rim was flailing extremely, and the patient was referred to surgery due to repeated implant device malposition and moderate degree residual flow. In the post-procedure control TTE, mild pericardial effusion (Peff) developed in one patient in the complex patient group, but there was no change in Peff size in the pre-discharge controls. It was observed that Peff disappeared completely in the first month. In 7 patients, mild-degree left–right residual flow was observed with TEE in the controls during the procedure after device implantation. Five patients showed mild residual shunting in the TTE performed after an average of 6 months and late-term after the occlusion. Three of these five patients had complex ASDs. Of these three complex ASDs, one patient had a defect that was very near to the IVC, and two patients had a defects that were not the aortic rim and flailing posterior rim. Two of the other patients with mild residual shunt had an ellipsoid-shaped ASD structure. No device-related tissue erosion, device embolism, device malposition, device thrombosis, cerebrovascular accident, cardiovascular event, additional thromboembolic complication, pulmonary embolism, cardiac or noncardiac mortality, or permanent cardiac arrhythmia was observed in the TTE controls and clinical follow-ups during the total follow-up period.

As a result of the measurements obtained with 2D and 3D TEE in circular and oval defects, no statistically significant difference was found in the comparison of minimum defect size and residual rims (*p* > 0.05). When comparing the measurements made with 2D TEE and 3D TEE in complex defects, statistically significant differences were found between the minimum defect size, aortic rim, and IVC rim measurements (*p* < 0.05). In complex defects, the minimum defect size, aortic rim, and IVC rim measurements are larger than 2D TEE measurements compared to 3D TEE measurements.

No statistically significant differences were obtained between other rims in complex defects according to measurement methods (*p* > 0.05) (Table 2).

**Figure 6:**
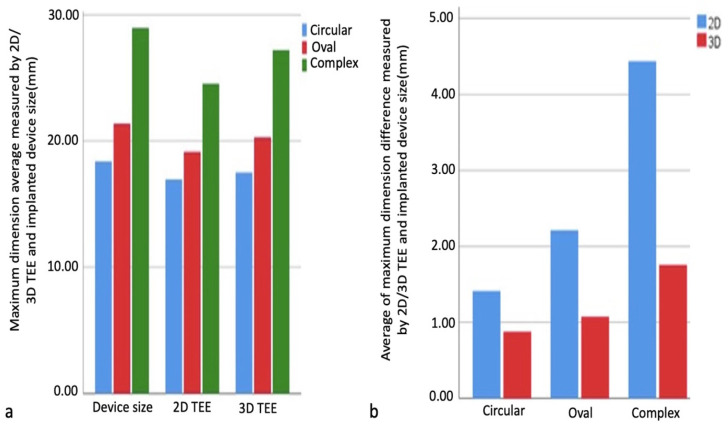
Comparison of maximum size measurements obtained by 2D and 3D TEE imaging and implanted device size. Comparison of maximum size measurements obtained by 2D and 3D TEE imaging and implanted device size in each type of defect (**a**). Comparison of the difference between maximum dimension measurements obtained by imaging methods and implanted device size (**b**). 2D, two-dimensional; 3D, three-dimensional.

No statistically significant differences were found in the comparison of maximum size measurements obtained by 2D TEE and 3D TEE imaging in all defect types (*p* > 0.05). In all defect types, the differences between the implanted device size and the maximum size measurements obtained from the 2D TEE and 3D TEE measurements were calculated, and change scores were found. Independent sample T and Mann–Whitney U tests were performed to compare change scores using measurement methods. The result of the analysis performed for the complex-shaped defect group showed statistical significance between the implanted device size and the differences in the maximum size measurement obtained from the 2D TEE and 3D TEE measurements (*p* < 0.05). The difference between the maximum size measured by 2D TEE and the implanted device size is greater than between the maximum size measured by 3D TEE and the implanted device size (Table 3). Comparison of ASD dimensions measured by 3D TEE/2D TEE and implanted device dimensions are shown in Figure 6a,b.

**Figure 7:**
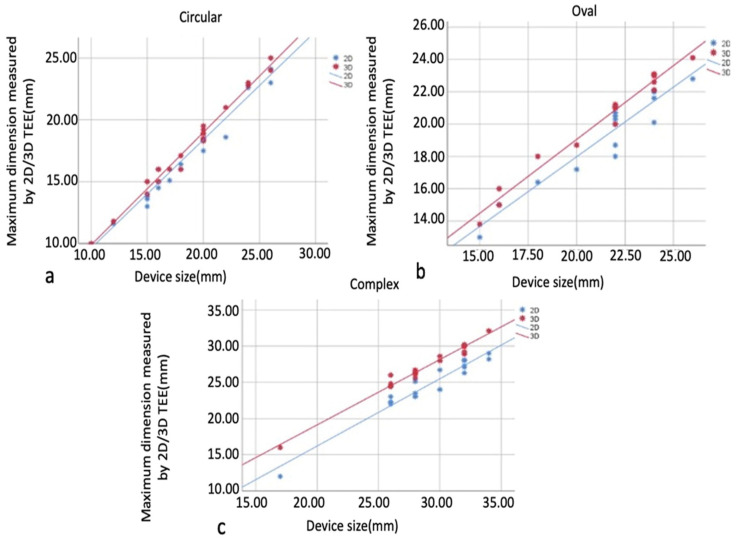
Graphs showing the correlation relationship between maximum size measurements and implanted device size in defect types. Circular defect and implanted device (**a**), oval defect and implanted device (**b**), complex defect and implanted device (**c**). In all groups, there is a high correlation between device size and 2D TEE and 3D TEE measurements, which is more significant in the 3D TEE group. 2D, two-dimensional; 3D, three-dimensional.

Pearson and Spearman’s correlations examined relationships between maximum size measurements and implanted device size within defect types. As a result of the analyses, correlation coefficients were observed in all 2D and 3D measurements obtained from circular, oval, and complex defects (0.982–0.992, 0.924–0.967, 0.946–0.973, respectively), and their relationship with the device size was statistically significant, positive, and meaningful (*p* < 0.05). There was a high correlation between device size and 2D TEE and 3D TEE measurements in all groups, and this correlation was found to be more significant in the 3D TEE group. The correlation relationship between the measured maximum size of circular, oval, and complex defects and the implanted device size is shown, respectively, in Figure 7a–c.

## 4. Discussion

Due to the techniques and devices developed for treating ASD, the transcatheter method is successfully applied with excellent effectiveness and a low complication rate [24]. When transcatheter closure is decided, it is very important to accurately determine the defect’s size, shape, and location to select the appropriate device. Because the atrial septum is extremely mobile during the cardiac cycle, it is sometimes challenging to determine the defect’s minimum or maximum diameter and morphology. In studies conducted on defect types, differences are observed in many parameters, such as residual rim and minimum and maximum defect sizes, depending on the measurement method [25]. The BS method is one of the methods used in determining the occluder device diameter; as a matter of fact, excessive stretching of the residuals during defect diameter measurement with BS may lead to the selection of a large-size device. Again, BS has some limitations and complications. Examples include septum rupture, cardiac rupture, balloon rupture, increased radiation exposure, and prolonged procedure time [26]. TEE is another frequently used method for defect size measurement and device size estimation. In studies comparing BS and TEE, it has been observed that BS is oversized when measuring some defect diameters, and TEE is useful for estimating the optimal device size. It was concluded that it is not necessary to routinely use BS in ASD closure in cases where TEE is used [8,27,28,29,30].

The 2D TEE and 3D TEE are frequently used methods in ASD closure, and 3D TEE imaging reveals the dynamic structure, spatiotemporal relationship, shape and size, relationship with surrounding tissues, and residual rim status of complex defects in great detail [31,32]. Data and comparisons of procedures and clinical outcomes using 3D TEE and 2D TEE are increasing [18,33]. In studies comparing BS, 2D TEE, and 3D TEE, the measurement results of the defect differ. It has also been observed that the defect’s shape affects the optimal device’s size. Although BS generally overestimates the device size, there are studies in which 3D TEE is correlated with BS and 2D TEE underestimates [17,18,25]. When deciding on device implantation in ASD closure, it is generally decided to choose a device 2–4 mm larger than the dimensions obtained from TEE measurement. This difference may vary further depending on the rim status of the defect, its flail, and the complex structure of the defect [25,33]. Formulas, indexes, and parameters have been created in some studies using 3D TEE to estimate device size. Using the indices and formulas created with these equations for all defects may cause measurement deviations in some defects and pose difficulties in routine clinical use. In addition, these parameters need to be applied or tested in different types of defects and large patient groups, and a gold standard method does not currently exist [6,15,33,34]. Our study was based on the comparison of size measurements in different types of defects with 2D TEE and 3D TEE. Choosing the optimal device for complex ASDs is still a big problem. According to the defect shape, our patients were divided into three groups (circular, oval, and complex). In 2D TEE measurements, scanning was performed according to the cardiac cycle, and the longest measurements were taken as basis. In diameter measurements with 3D TEE, the MPR method, known for its practicality and clinical reliability, was used [6,18,25]. In this and similar studies, where defect sizes were evaluated with MPR in 3D TEE and compared with other imaging modalities, a significant correlation was determined between the maximum-measured diameter and the size of the implanted device [6,18,34]. Again, in a retrospective study comparing 3D MPR sizing with conventional measurement methods between BS and 2D TEE, the defect diameter measurement was found to be highest in BS and smallest in 2D TEE, and also in complex-shaped patients with increased elliptic index and increased high aneurysm amplitude [14]. In our study, in the 2D TEE and 3D TEE measurements of circular and oval-shaped defects, although the length averages obtained from 3D TEE measurements were higher in the minimum and maximum diameters of these defects and the sizes of their residual rims, no statistical significance was revealed due to the measurement difference in these groups (*p* > 0.05). Similarly, in studies, maximum size measurement in circular and oval defects was similar between groups, and a correlation was observed between other measured defect diameters [6,15,18,33]. In our study, as the defect type became more complex and the defect size increased, measurement differences emerged between the 2D TEE and 3D TEE measurements and residual sizes. As a result of the measurements in complex defects, statistical significance was observed in the comparison of the differences between the selected device size and the defect size measured by both methods (*p*: 0.025). The selected device size was decided on according to the measurement result (based on the maximum size) obtained with the 3D TEE. There is a correlation between both measurement methods and the size of the implanted device, and this correlation was observed stronger in the 3D TEE. Differences emerged in the 2D TEE and 3D TEE in evaluating the residual rims of complex defects. In our study, in TEE measurements made in complex defects, IVC and aortic rim size were observed to be larger in measurements made with 3D TEE, which showed statistical significance (*p* < 0.05). Naturally, the data supporting the measurement results we obtained in our study are available in the literature. In this and similar studies, 3D TEE measurements were larger than those of 2D TEE in complex-shaped ASD. Additionally, septal residual rims are more clearly defined with 3D TEE than 2D TEE [17,18]. Three-dimensional TEE imaging support provides remarkable information in percutaneous interventions to treat complex ASDs [22]. In general, our study observed that as the defect size and complexity increased, the difference between the measurement results with 2D TEE and 3D TEE increased. Our study observed a high correlation between the implanted device and the measurement values, more so in the 3D TEE measurement. In complex ASDs, differences in diameter and area are much larger between different TEE measurement methods due to localization and/or complex shape. In addition, in standard 2D TEE, cross-orthogonal cross-sectional planes generally do not exactly cut the minimum or maximum distance from the defect to the surrounding residual rim tissues. For this reason, rim lengths may also give incorrect results. Therefore, deciding alone in borderline cases or complex defects may be difficult. This incorrect prediction can be prevented using detailed examinations obtained by cropping the 3D TEE datasets covering the ASD and the surrounding residual rim structures [11]. In particular, showing the IVC rim with 2D TEE cannot be easy because it is distant in the acoustic field. However, with the help of real-time 3D TEE imaging and 3D TEE-cropping software (QLAB version 10.5), the IVC residual rim and aortic residual rims can be shown much more clearly. Therefore, in our study, IVC and aortic residual rims measured by 3D TEE were statistically larger in the complex ASD group. Three-dimensional TEE allows a safer selection of the size of the occlusion device, especially in patients with ASDs close to the IVC and with borderline or absent aortic rim. Three-dimensional TEE can easily determine whether the defect rim is loose or rigid, which helps operators rationally adjust the device size [11]. In our study, the patient with complex ASD, no aortic rim, excessively flaccid inferior rim, and aneurysmatic septum was referred to surgery as the device malapposed in repeated implantations, and advanced residual flow was observed. No serious complications were observed in our patients in the intraoperative and postoperative periods and the TTE controls and clinical follow-ups during the total follow-up period. No device-related tissue erosion, device embolism, device malposition, device thrombosis, cerebrovascular accident, cardiovascular event, additional thromboembolic complication, pulmonary embolism, or cardiac and no noncardiac mortality or permanent cardiac arrhythmia were observed. Initially, a mild residual shunt was observed in seven patients, but the amount of shunt and the number of patients with shunt decreased during the follow-up. Naturally, it has been observed that after ASD closure, slight and trace amounts of residual flow can disappear with gradual endothelialization [18]. Our periprocedural success and clinical outcomes correlate with studies comparing similar methods, are clinically applicable, and are supported by the literature [8,18,25,28]. Of course, fluoroscopy and other imaging methods have also contributed to the success of this procedure and are an integral part of the procedure to achieving optimal results. Many imaging methods are now used together in the diagnosis, risk level determination, and treatment of congenital, structural, and cardiovascular diseases. With this multimodal imaging, especially 3D TEE, appropriate patient screening, procedure indication, pre-procedure risk assessment, and treatments are implemented safely and effectively [35,36].

In our study, 3D TEE appears to be a very safe and feasible method for successful closure of complex ASDs. The anatomical structures of these defects, their relationship with the surrounding tissues, the status of the remaining rims, and the compatibility of the selected device size with the defect size were determined more accurately with 3D TEE. The absence of negative outcomes in the perioperative and late periods shows the importance of 3D TEE guidance in the management of patients with complex ASD and other congenital defects. While cardiac imaging technology is developing rapidly around the world, not all cardiology departments may have the current technological tools. This situation negatively affects both the operator experience and the optimal level of the treatment process. In order to better continue this learning process, it is undoubtedly important to share more comprehensive data obtained from different patient groups.

## 5. Conclusions

In our study, 3D TEE and 2D TEE measurement results and implanted device size were highly correlated in circular and oval defects. In complex defects, a significant difference was observed between the implanted device size and the maximum defect size obtained from the 2D TEE and 3D TEE measurements. The 2D TEE under-estimated defect diameters, IVC, and aortic residual rims in complex ASDs. The 3D TEE has been seen as a very safe and feasible method for successfully closing defects in patients with complex ASD (the anatomical structures of the defect, its relationship with the surrounding tissue, and the status of the residual rims are well-defined with 3D TEE). As a result, while ASD implantation can be performed successfully with 2D TEE and 3D TEE in circular and non-complex oval defects, 3D TEE measurements and 2D TEE measurements showed differences in the procedures performed in complex ASDs, and 3D TEE showed a higher correlation with the device size. In complex ASDs, closing the defect with only 2D TEE may lead to selecting a smaller-sized device. As 3D TEE becomes more widespread, operators will better understand the three-dimensional anatomy of ASD. Thus, the selection and implantation of the appropriate closure device will become more manageable, and complications will be minimized.

### Limitations

Our study is retrospective. The limitation of our study was the small number of patients included in the analysis, but the number of patients is still compatible with similar publications in the literature. Another limitation is that only one type closure device was implanted in the patients.

## Figures and Tables

**Table 1:** Clinical characteristics and demography of the patients.

Age/year (mean ± standard deviation)	35.05 ± 13.87
Follow-up period/month(mean ± standard deviation)	15.30 ± 9.18
Women (*n*, %)	41 (67.2)
CAD (*n*, %)	7 (11.4)
DM (*n*, %)	5 (8.19)
AF (*n*, %)	2 (3.2)
HT (*n*, %)	8 (13.1)
TIA/Stroke (*n*, %)	4 (6.55)
PH (*n*, %) (MPAP > 20 mmHg via RHC)	26 (42.6)
Symptoms with RHD (*n*, %)	48 (78.6)
Symptoms without RHD (*n*, %) (Qp/Qs > 1.5)	9 (14.7)
RHC (*n*, %)	38 (63.9)
COPD (*n*, %)	5 (8.19)
CKD (*n*, %)	4 (6.55)

CAD, coronary artery disease; DM, diabetes mellitus; AF, atrial fibrillation; HT, hypertension; TIA, trans-ischemic attack; PH, pulmonary hypertension; RHD, right heart dilatation; COPD, chronic obstructive pulmonary disease; CKD, chronic kidney disease; MPAP, mean pulmonary artery pressure; RHC, right heart catheterization.

**Table 2:** Comparison of residual rim dimensions and minimum defect dimensions obtained by 2D and 3D TEE imaging in each type of defect.

Circular				Oval		Complex	
Residual Rims (mm)	Imaging	Mean ± SD	*p*	Mean ± SD	*p*	Mean ± SD	*p*
AV valve	2D	9.36 ± 2.51	0.550	7.04 ± 2.04	0.259	5.97 ± 0.91	0.626
	3D	9.80 ± 2.70		7.45 ± 1.66		6.12 ± 1.02	
Aortic	2D	7.18 ± 2.51	0.563	5.21 ± 2.66	0.540	0.95 ± 1.67	0.012
	3D	7.47 ± 2.79		5.81 ± 2.34		2.29 ± 2.23	
SVC	2D	14.63 ± 3.68	0.680	13.45 ± 3.31	0.683	12.90 ± 1.54	0.115
	3D	15.08 ± 3.91		13.94 ± 3.65		13.21 ± 1.65	
IVC	2D	10.27 ± 1.88	0.695	8.15 ± 1.77	0.215	6.66 ± 1.11	<0.001
	3D	10.48 ± 1.92		8.94 ± 1.88		8.31 ± 0.99	
Posterior	2D	12.74 ± 3.03	0.422	11.61 ± 2.58	0.586	10.82 ± 2.49	0.121
	3D	13.26 ± 3.21		12.12 ± 2.77		12.13 ± 2.24	
Min ASD Dimension (mm)	2D	14.51 ± 2.52	0.733	13.18 ± 2.52	0.091	18.77 ± 3.45	0.037
	3D	14.15 ± 3.21		11.85 ± 1.88		21.18 ± 3.57	

AV, atrioventricular; SVC, superior vena cava; IVC, inferior vena cava; Min, minimum; 2D, two-dimensional; 3D, three-dimensional; SD, standard deviation.

**Table 3:** Comparison of maximum size measurements obtained by 2D and 3D TEE imaging and implanted device size in each type of defect.

Defect Type	Imaging and Device	Mean ± SD	*p*
Circular	Device size (mm)	18.38 ± 4.12	0.446
	2D size (mm)	16.96 ± 3.68	
	3D size (mm)	17.50 ± 3.78	
Oval	Device size (mm)	21.35 ± 3.26	0.380
	2D size (mm)	19.14 ± 2.92	
	3D size (mm)	20.28 ± 3.01	
Complex	Device size (mm)	28.95 ± 3.86	0.069
	2D size (mm)	24.52 ± 3.72	
	3D size (mm)	27.20 ± 3.53	
Comparison of the difference between maximum dimension measurements obtained by imaging methods and implanted device size.			
Defect type	Device size-measured dimension	Mean ± SD	*p*
Circular	Device-2D (mm)	1.41 ± 0.86	0.549
	Device-3D (mm)	0.88 ± 0.60	
Oval	Device-2D (mm)	2.21 ± 0.93	0.170
	Device-3D (mm)	1.07 ± 0.55	
Complex	Device-2D (mm)	4.44 ± 1.04	0.025
	Device-3D (mm)	1.76 ± 0.65	

SD, standard deviation; 2D, two-dimensional; 3D, three-dimensional.

## Data Availability

The data that support the findings of this study are available from the corresponding author upon reasonable request.
